# Cholera Infantum

**Published:** 1891-12

**Authors:** William Steinrauf

**Affiliations:** St. Charles, Mo.


### CHOLERA INFANTUM.

WILLIAM STEINRAUF, M. D., ST. CHARLES, MO.

The longer I live and practice medicine, the more I am being convinced that high potencies exert the best of influence in all curable cases of disease, whether acute or chronic. I know there is a talk amongst some of our physicians of giving "appreciable doses of medicine," of "administering drugs that will make an impression," etc., but I also know from experience that all such talk is based either on ignorance as regards the high potencies, or is generally indulged in by such physicians who favor a mixture of Homœopathy and Allopathy, thus rendering deep thought and constant study of their cases unnecessary.

I know it is much easier to give Quinine against fever, Morphine to check pain, cathartics to open the bowels, and astringents to check diarrhœa, than to thoroughly study the case and give the remedy indicated by the symptoms, according to the rules of Homœopathy laid down by the immortal Hahnemann. I know well how hard it is for a conscientious homœopathic physician doing pioneer or missionary work in some parts of our country to strictly follow Hahnemann's plan, how he is being daily tempted to deviate from what the fathers taught us. Why? Simply because the great mass of the people know nothing of Homœopathy. Take this State of Missouri. There are, comparatively, very few physicians of our school in the State. The homœopathic physician, especially if he is alone in a place where he has to grabble with a half dozen allopathists, that can hold his own and practice pure Homœopathy must indeed be a hard student. Nothing but hard work will help him. Nothing but toil, toil day and night, in season and out of season, will save him from drowning in the mire of eclecticism.

It is somewhat different where several homœopathic physicians labor in one place, the one becomes an example for the other, they can come together to consult, one exerts an influence over the other. It is easier, all things being equal, to be a better homœopathist where this is the case. According to Hoyne's *Homœopathic Directory*, where he gives the progress made in our school in the different States for the last few years, the *Directory* says that even in Missouri, " where little was expected," there had been quite an accession to our practice from the laity. The great trouble with us in Missouri—and it is the same in many other places—is that many of our physicians do not practice Homœopathy. Allopathists are continually holding us up to the ridicule of the people, by showing them that we are not true to our principles. Let us cease reading old-school writings and study Hahnemann's *Organon*, *Chronic Diseases*, and *Materia Medica*, and thus become healers in spirit and in truth.

These thoughts occurred to me during the past few weeks whilst cholera infantum was prevalent amongst the little ones here in our city. I have only used the high and highest potencies, and of great numbers treated only one has died. And this was a two-months'-old baby, was being fed by bottle, and died of marasmus. The remedies mostly used were:

*Apis.* The child is inclined to stupor, out of which it starts with a loud, shrill scream. The eyes look red and the head is hot. Although the mouth and tongue are dry, there is seldom much thirst. Skin is quite dry. Suppression of urine. The diarrhœa is worse in the morning and generally mixed with mucus.

*Arsenicum.* Diarrhœa and vomiting; much thirst for cold water, but everything the child drinks is thrown up at once. The skin is hot and there is great restlessness. The child continuously moves and cries incessantly. Stools are watery and very offensive. There is great weakness and emaciation.

*Belladonna.* This remedy did splendid work, and was indicated in about nine out of every ten cases. Where the cases could be seen in the very start it would be the only remedy required. Tho child lies in a stupor, it frequently starts up suddenly in its sleep. When awake it is angry and violent. The face is generally red and hot, at times cold and pale. Hands and feet cold; the abdomen is hot. The pulse is frequent and feels as if a shot were passing under the finger. The stools are clay-colored, green, or consist of mucus.

*Chamomilla.* The child is very peevish. The gums are very hot and the little patient wants to be carried all the time. There are colicky pains and vomiting. The passages are green or green mucus, looking like chopped eggs and smelling like rotten eggs. The discharges are hot and excoriate the parts.

*Ipecacuanha.* Diarrhœa and vomiting. Vomiting predominates. Much nausea; face pale and oppressed breathing. Stools are green, bloody, and fermented.

*Nitric-acid.* Putrid smell from mouth; copious flow of saliva; ulcers in mouth and tongue.

*Podophyllum.* The diarrhœa is worse in the morning, and the discharges are more frequent at night than during the day. The stools are green, watery, or look white like chalk; profuse and painless. Very often prolapsus ani. Often have cough and catarrh of the chest.

*Sulphur.* In desperate cases. Hands and feet cold the very first morning. The child lies in a stupor and there is entire suppression of urine. Here one or two doses of Sulphur would bring about reaction and a speedy change for the better.

*Veratrum-alb.* Was indicated a few times only. During stool cold perspiration on the forehead. Voice weak or hoarse.

*Oleum-ricinus* in the DMM was given in a number of cases that were treated by mail. The descriptions were so vague that it was next to impossible to find the simillimum. In some cases a cure resulted, and in others there was a decided change for the better. I know of no proving.

The diet needs strict attention, all the water the little patient craves, and a flannel bandage over the abdomen. This is worn all summer.

